# Outcomes of a Proximal Workplace Intervention Against Workplace Bullying and Harassment: A Protocol for a Cluster Randomized Controlled Trial Among Norwegian Industrial Workers

**DOI:** 10.3389/fpsyg.2020.02013

**Published:** 2020-08-31

**Authors:** Kari Einarsen, Morten Birkeland Nielsen, Jørn Hetland, Olav Kjellevold Olsen, Lena Zahlquist, Eva Gemzøe Mikkelsen, Justine Koløen, Ståle Valvatne Einarsen

**Affiliations:** ^1^Department of Psychosocial Science, University of Bergen, Bergen, Norway; ^2^Department of Leadership and Organizational Behaviour, BI Norwegian Business School, Bergen, Norway; ^3^National Institute of Occupational Health, Oslo, Norway; ^4^Department of Psychology, University of Southern Denmark, Odense, Denmark; ^5^Kvaerner AS, Stord, Norway

**Keywords:** workplace bullying, harassment, prevention, bystander behavior, intervention, Grip inn

## Abstract

**Background:** Workplace bullying is an important and prevalent risk factors for health impairment, reduced workability and lowered efficiency among both targets and observers. Development and tests of effective organizational intervention strategies are therefore highly important. The present study describes the background, design, and protocol of a cluster randomized controlled trial evaluating the effectiveness of an organization-wide intervention on preventing workplace bullying with a focus on promoting active and constructive bystander behavior. The main overarching goal is to develop an easy to use and standardized organizational intervention based on theory and research in the role of bystanders in bullying situations with the potential of reducing the prevalence of workplace bullying. The theoretical framework of the study is theory of planned behavior (TPB; Ajzen, 1991).

**Methods/Design:** Using a full randomized control trial (RCT) design, this project will empirically test the outcomes of an intervention program targeting bullying and harassment as the main distal outcomes and perceived behavioral control and helping behavior among bystanders as the main proximal outcome. A 1-year cluster randomized controlled design will be utilized, in which controls will also receive the intervention. About 1,500 workers from two different locations of a Norwegian industrial company will be randomized into one intervention group and two control groups with at least 400 workers in each group. A survey will be conducted electronically. With a total of three assessments over 10–12 months, the time interval between the measurement times will be 4 months. Thus, the data collection will take place at baseline, completion of the intervention and at 4 months follow-up.

**Discussion:** This study primarily aims to develop, implement, and evaluate an intervention based on the abovementioned features with the ultimate aim of reducing the prevalence of workplace bullying, by awareness raising and training of bystanders. Manager involvement and involvement of the union representative and the elected health and safety representatives is an important feature of the program. Results of the intervention study will provide important information regarding the effectiveness of preventive interventions against workplace bullying when focusing on bystanders, particularly so regarding the role of bystander awareness, bystander self-efficacy, and bystander behavioral control on the one hand and the prevalence of bullying and harassment on the other.

## Introduction

Workplace bullying and harassment is a prevalent problem in contemporary workplaces all around the globe (van de Vliert and Einarsen, 2014; Zapf et al., 2020), with documented devastating effects on the targets health and well-being (Niedhammer et al., 2012; Lesuffleur et al., 2014; Nielsen and Einarsen 2018; Jacobsen et al., 2020; Mikkelsen et al., 2020). The concept of workplace bullying and harassment refers to a systematic form of aggression and social exclusion where an employee, persistently and over a period of time, is exposed to negative actions from superiors or coworkers where the employee finds it difficult to defend himself/herself against the aggression (Olweus, 1993; Einarsen et al., 2020). Synthesized findings from systematic reviews and meta-analyses of cross-sectional and longitudinal studies show that targets of such treatment have an elevated risk of mental and somatic health problems (Nielsen and Einarsen, 2012; Nielsen et al., 2014; Verkuil et al., 2015), including symptoms of posttraumatic stress (Nielsen et al., 2015) and long term sickness absence (Nielsen et al., 2016). While individual targets even risk exclusion from working life (Glambek et al., 2016), organizations risk productivity loss, loss of reputation, and even financial losses (Hassard et al., 2017; Hoel et al., 2020).

With an estimated global prevalence rate of up to 15% of more severe cases (Nielsen et al., 2010), the development of effective preventive efforts is paramount. However, to this date, few interventions have been tested empirically and few have been conducted with high quality research designs. Furthermore, those interventions that have been properly examined in research have mainly been beneficial with regard to reducing aggressive behavior of low intensity, such as incivility, whereas they have been unsuccessful and ineffective with respect to more severe cases of workplace bullying (Hodgins et al., 2014; Escartin, 2016; Gillen et al., 2017).

An explanation for why most previous attempts at interventions toward workplace harassment bullying have failed may be that the measures undertaken have been all-encompassing interventions focusing on the more broad issue, such as respect, dignity, and civility, or on psychosocial work environment factors in general, rather than explicitly addressing bullying and harassment (Probst et al., 2008; Einarsen et al., 2009; Osatuke et al., 2009). Hence, most interventions have been general rather than selective and indicative, as in the case in the present study. Hence, an intervention that aims to reduce the occurrence of workplace bullying should have a clear focus on specific wanted and unwanted conduct, in our case by directly addressing bystanders behavior when observing acts of bullying at work. Since most cases of workplace bullying escalates over time with targets being exposed to varying degrees of interpersonal aggression – from occasional exposure to negative acts of mere incivility to being severely victimized on a daily basis (Einarsen et al., 2020) – preventive interventions should address bullying in its early phases.

Another explanation for why previous interventions have failed is that organizational interventions are usually determined to be effective based on whether exposure to an intervention condition preempted a statistically significant improvement in a targeted distal outcome. However, according to Biggs and Brough (2015), several reviews have concluded that the success of organizational interventions is largely determined by facets of the context in which they are implemented (i.e., intervention context), how they are implemented, and to whom they are implemented (i.e., intervention process). When evaluating interventions, it is therefore important also to evaluate mediating proximal effects that are theoretically linked to targeted distal outcomes, that is, factors that hinder or facilitate the main outcome of the intervention (Nielsen and Abildgaard, 2013). In the case of workplace harassment and bullying, bystander behavior and bystander intervention has been proposed as a specifically important proximal variable with regard to the further development of workplace bullying (D’Cruz and Noronha, 2011; Pouwelse et al., 2018; Ng et al., 2019). Addressing the role of bystanders may therefore be beneficial when crafting an effective intervention. Specifically, by promoting social support and constructive behaviors among bystanders (mediating proximal effects), one may be able to also reduce the overall occurrence of workplace harassment and bullying in the longer run. Hence, the underlying intervention theory assumes a mediated process, whereby perceived or actual changes to distal outcomes (i.e., reduced occurrence of harassment and bullying) occur due to perceived or actual changes in proximal outcomes (bystander intervention).

A third explanation for why previous interventions toward harassment and bullying have failed is that they have not been conducted with sufficient methodological rigor to contribute to the evidence base for addressing the problem of workplace bullying and incivility (Escartin, 2016; Gillen et al., 2017). According to Gillen et al. (2017), there is presently only very low-quality evidence that organizational and individual interventions may prevent harassment and bullying in the workplace. Hence, there is a need for large well-designed controlled trials of preventive interventions employing both distal and proximal factors, preferably with a randomized control trial (RCT) design.

### Theoretical Model for the Intervention

The present study builds on the overarching proposition that having bystanders intervene more often and more constructively in everyday bullying and harassment episodes, preferably before the bullying situations have escalated, should help to reduce the occurrence and prevalence of this pertinent problem over time. The present intervention study then builds on the theory of planned behavior (TPB; Ajzen, 1991) to achieve this. The TPB proposes three determinants of a person’s intentions, behavior, and any following behavioral changes, attitude, subjective norms, and perceived behavioral control. These three factors will be seen as proximal and mediating outcomes, while observed and perceived exposure to bullying, including observed social climate, will be the more distal and final focal outcome. In our context, attitude will be addressed by the very nature of the intervention, that is, creating awareness and changes in attitudes regarding the existence and nature of workplace bullying and one’s own attitudes and perceived typical bystander behavior in such situations. The intervention is further designed to alter participants’ subjective norms by increasing the perceived social pressure to perform constructive bystander behavior in such situations. Lastly, by showing when and how bystanders may intervene constructively (Ajzen and Madden, 1986; Ajzen, 1991), the intervention should alter individuals’ perceived behavioral control in specific situations, the latter implying the extent to which the behavior is perceived to be under volitional control. Thus, developing a psychosocial climate in the organization where individual attitudes and social norms foster constructive bystander behavior, while also enhancing the employees’ perceived behavior control as bystanders, is hypothesized to be crucial in combating workplace bullying in organizations.

Accordingly, in addition to investigate any changes in bullying and harassment in the working environment, the present study will examine whether attitudes, social norms, and perceived behavioral control of those who have received an intervention called “Intervene,” will be changed and possibly influence bystander behavior in bullying situations. Ultimately, the intervention should reduce the occurrence of bullying and harassment and improve the social climate at work. As such, we hypothesize the following regarding these proximal outcomes:

1.Those who have received the intervention will have a change in **outcome variables (behavior)** from T1 to T2 as compared to those in the control group:
1.1.Those who receive the intervention will have an increase in *observed workplace bullying* from T1 to T2 as compared to those in the control group due to an increased awareness.
1.2.Those who receive the intervention should report lower exposure to bullying behaviors, as compared to those in the control group.
1.3.Those who receive the intervention will be more likely to *intervene when observing workplace bullying* from T1 to T2 as compared to those in the control group.
2.Those who receive the intervention will have a change in **independent variables (attitudes, norms, perceived control, and social climate)** from T1 to T2 as compared to those in the control group:
2.1.Those who receive the intervention will have an increase *in their attitudes toward intervene constructive when observing workplace bullying* from T1 to T2 as compared to those in the control group.
2.2.Those who receive the intervention will have an increase in *perceived bystander norms* from T1 to T2 as compared to those in the control group.
2.3.Those who receive the intervention will have a more positive view of the *social climate* in the working group from T1 to T2 as compared to those in the control group.
2.4.Those who receive the intervention will have an increase in perceived *informal surveillance* from T1 to T2 as compared to those in the control group indicating an increase in social anti-bullying norms in the working environment.
2.5.Those who receive the intervention will have an increase in *perceived bystander behavior control* from T1 to T2 as compared to those in the control group.
3.From T2 to T3 the control group, which will receive the intervention after T2, will show the same results of the abovementioned hypothesis as the intervention group did from T1 to T2.

**Figure 1:**
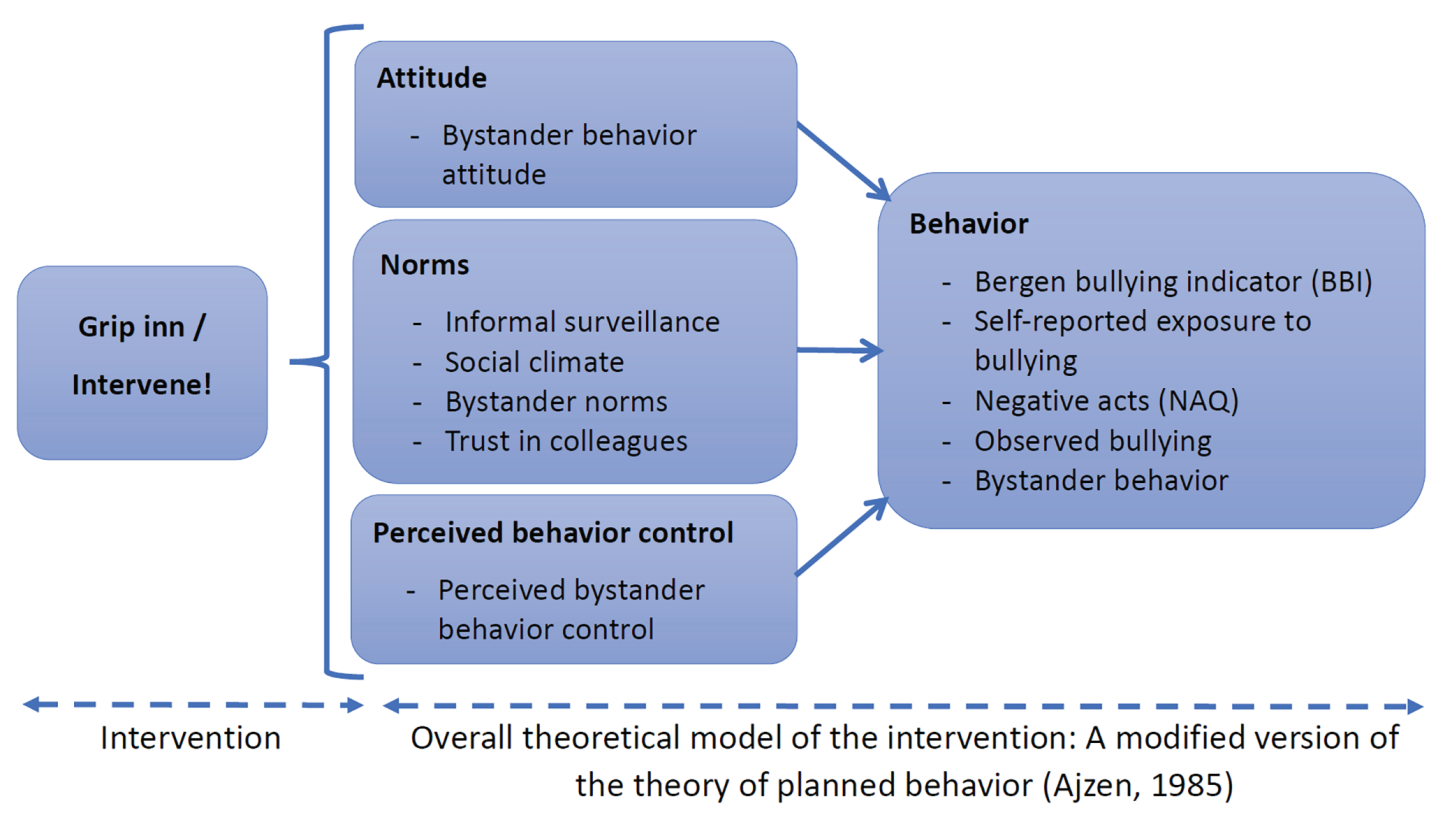
The theoretical model for the intervention is based on a modified version of the theory of the planned behavior (TPB; Ajzen, 1985).

An overview of the relationships studied in this intervention is given in Figure 1.

## Objectives

Using a full RCT design, the aim of this project is to develop and empirically test an intervention program targeting harassment and bullying as the main distal outcomes and helping behavior among bystanders as the main proximal outcome. While all occupational groups are potentially at risk for workplace harassment and bullying, some occupations are more exposed than others. Research in the Norwegian context (Einarsen and Raknes, 1997; Nielsen et al., 2012) as well as internationally (Baillien et al., 2011; Notelaers et al., 2011) has identified industry workers as a particularly risky group, and particularly so for low intensity bullying (Einarsen and Raknes, 1997). The intervention will therefore be conducted in a large Norwegian industrial organization that provides engineering, procurement, and construction services to the oil and gas industry. The oil and gas industry is a key driver for the Norwegian economy and knowledge about how reducing the occurrence of workplace harassment and bullying may therefore have important implications at the individual, organizational, and societal levels. This protocol will describe the trial design, participants, procedure, intervention program elements, randomization, ethical consideration, and statistical analysis that will be included in the project.

## The Intervention

This intervention is based on an existing workplace intervention developed by researchers and practitioners in Denmark, yet tailor-made for the public sector (Mikkelsen and Hogh, 2019). The intervention was customized to a Norwegian industrial setting by the authors, representing both researchers and practitioners. Consequently, the intervention described here is based on the structure of the original Danish intervention but is customized to a new sociocultural, industrial, and socioeconomic context. It should be noted that in accordance with the original intervention, a main overarching goal of this research is to develop an easy to use and standardized intervention which enable user-organizations to master the implementation themselves, and thereby not be dependent on professional and external consultants.

**Figure 2:**
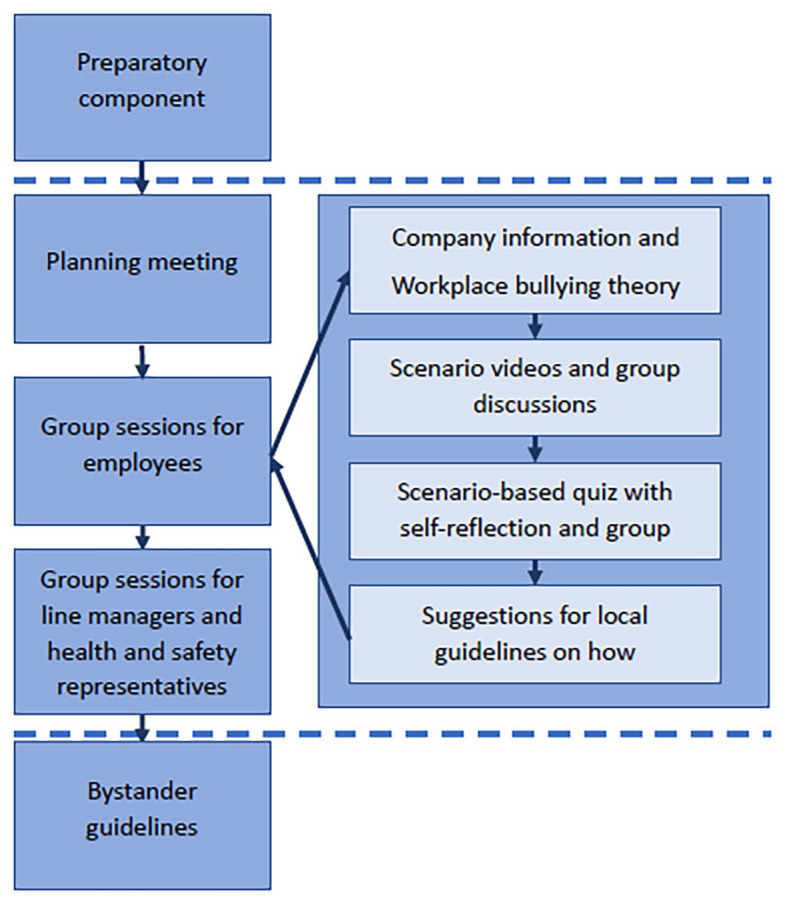
A description and flow-chart of the contents and main elements of the intervention.

The workplace intervention, called “Grip inn” (“Intervene!”), is based on existing literature and empirical research on bystander behavior (e.g., Paull et al., 2012; Pouwelse et al., 2018; Ng et al., 2019). The main theoretical assumption behind the intervention is that the actions of bystanders in situations where coworkers are exposed to acts of bullying and harassment can either escalate or reduce workplace harassment and bullying. Hence, stimulating early, active and constructive intervention by bystanders should prevent further escalation and possibly create an anti-bullying climate. A flow-chart of the content of the intervention is shown in Figure 2 and will be described in the following.

The intervention comprises a preparatory part in order to anchor the program among managers, health and safety representatives, and labor union representatives. This initial preparatory part aims at increasing ownership to the program at all management levels and among elected health and safety representatives, and labor union representatives, thereby ensuring that key stakeholders contribute to successful implementation and follow up of the intervention.

The program is further divided into **three elements:**

1. A **planning meeting** at the workplace/department prior to the program with managers at the department level, the local labor union representative, the local health and safety representative, and a local HR representative.
2. A 3-h **group session** with work-teams of 15–30 employees with their supervisors, managers, and elected health and safety representatives as participants.
3. A **follow-up session** with department managers, those line managers, and elected health and safety representatives that participated in element two with the aim of following up ideas and suggestions for bystander guidelines created by employees in element two.

These three elements will now be described in more detail:

The first element consists of an initial meeting with the department manager and HR representatives laying the foundation for how to divide the department into groups used in the second part of the program and to prepare the department manager for his or her active role as host in the main intervention.

The second element and main part of the intervention, the group sessions, concentrates on the participants’ awareness of, knowledge about, and perceptions related to workplace bullying and harassment and of the possible role of bystanders. The main focus is on the opportunities for bystanders to intervene constructively in such situations. The sessions have a duration of 3 h and are divided into four consecutive parts with short breaks between each section.

1. An opening part is led by the department manager, starting with a 5-min video talk by the executive vice-president on the importance of the intervention to the company. Thereafter, the department manager provides a talk on the importance of the issue, providing also information about the organization’s policies and procedures for handling workplace bullying and its complaints and investigation procedures. Thereafter, the consultant leading the intervention has a 15–20-min presentation on the issue of workplace bullying and the role of bystanders and their possible role taken in a bullying scenario. This part is intended to last for 45 min.
2. The second part is made up of two 10 min films describing two typical bullying scenarios, each followed by group discussions on the role of the bystanders in these films and how the bystanders intervened and could have intervened more constructively than they actually did. This part adds up to approximately 40 min.
3. The third part includes a scenario quiz focusing on possible bullying and harassment situations asking how participants themselves think they would have handled the situation (by using an interactive presentation tool), followed by group discussions on how their work team usually reacts and should react to these different kinds of examples of bullying and harassment. This part is intended to last for 40 min.
4. The last part consists of a group discussion with the aim of developing suggestions and norms for how one should react as a bystander and how one may intervene in specific situations with bullying and negative acts. Each group writes up their suggestions and ideas. This last part is intended to last for about 30 min, including a closing talk from the department manager of the way forward, the importance of the issue at stake, and his/her summation of the 3-h session.

The third element is a group session for line managers and a health and safety representative. The aim of this session is to develop local guidelines for the department on how bystanders could and should intervene when observing acts of bullying and harassment, based on the suggestions derived from the “Grip inn/Intervene!” group sessions. Lastly, an action plan for the implementation and follow up of these bystander guidelines are discussed and developed. The session will be led by the department manager and a consultant.

### Basic Principles in the “Grip Inn/Intervene!” Group Sessions

The “Grip inn/Intervene!” program is based on three pillars. A fundamental approach in all three pillars is participant involvement (Birzer, 2002). As a first pillar, scenario-based training will be introduced in order to increase bystander awareness, knowledge, and behavior. The second pillar will involve a scenario-based quiz that is developed in order to increase the knowledge of how bystander roles may relate to their own work context and to make the participants reflect on their own bystander behavior as well as the behavior of their work team. As the third pillar, the work teams develop suggestions for how bystanders should act, which is further developed into local guidelines in a collaboration including the manger and the local elected health and safety representative. This is done to ensure formal commitment and further enhancement of bystander intervening behavior and to engage and place the intervention within ongoing formal labor relations and health and safety system in the said organization.

Furthermore, the intervention assumes that upper management support and active involvement and ownership among department managers are crucial to success, hence role of the department manager and the executive vice president are vital in the planned intervention.

## Methods and Analysis

### Reference Group

In order to establish the intervention in the case company and to calibrate the perceptions of the research group with the actual working environment in the company, a reference group is set up to advice on the implementation of the project. The reference group is comprised of key stakeholders within the case company. The members were recruited by the HR department of the case company and include upper managers, first line managers, an occupational health representative, labor union representatives, and health and safety representatives.

### Study Design

This project has an RCT design that includes a range of departments from the case company, a Norwegian industry company. The company has two main locations, one on the west coast (location A) being the larger plant with some 1,600 employees and one in mid Norway (location B) being the smaller plant with some 800 employees. In location A, being the larger facility, all departments in the production part and two administrative departments will be randomly assigned to an intervention group and a control group. The control group will receive the intervention 6 months later, as will location B. As such, the location that is given the intervention last (location B) will also serve as an additional control group for the first interventions carried out in location A. Hence, we have two control groups for the initial intervention. In order to carry out a valid and reliable study of causal relationships and mechanisms, it is necessary to use a design where the study variables are measured repeatedly over time. In this project, we will use a mixed method design by combining a longitudinal survey, qualitative interviews, with this RCT design. All employees in the selected departments in the two locations of the case company will be invited to participate in a prospective questionnaire survey that will be distributed three times during a 12-month period. The questionnaire will be identical at all assessment points. It will be possible to distinguish between managers with personnel responsibility, managers without personnel responsibility, safety representatives, labor union representatives, other level employees, and the unit to which they belong. Data will be collected through *Surveyexact*, a web-based system for secure administration of questionnaires. The system is developed for the purpose of tracking individuals over time in a way that satisfies demands for anonymity and personal security and data protection. When accessing the web-based questionnaire by a link, the respondents will be asked to provide their informed consent before responding to the questionnaire. No personally identifiable information about respondents will be available to researchers, as data will be de-identified prior to analyses.

To obtain detailed information on how the intervention is perceived by the participants, qualitative interviews will be conducted with selected employees in different units/teams of the case company. Finally, immediately after the intervention, a short questionnaire and evaluation form focusing on satisfaction and perceived relevance of the intervention program will be sent out to all those who participate in the 3-h-group-sessions of the intervention.

### Sample

All employees in the intervention and control groups will be invited to participate in the three survey measurements. The total number of employees participating from both locations amounts to about 1,500. The surveys will be carried out approximately 4–6 months after intervention as well as 10–12 months, this to study the outcomes of the control groups who receive the intervention 6 months later and in order to study more long-term effects of the initial intervention.

Each employee in the company is provided with a smartphone when starting their employment. The survey will therefore be conducted electronically using these personal smartphones. The purpose of the survey will be to identify changes in the prevalence of bullying behavior, the social climate in the department, as well as the role played by bystanders in the working environment.

### Statistical Power

In order to estimate sample size, we conducted a power analysis. One of the primary goals of power analyses is to estimate the number of respondents required in a study to minimize the likelihood of a false negative finding. In the context of intervention studies, the goal is to estimate the number of respondents per group to detect a difference between intervention and control groups. An *a priori* power analysis was conducted using G*Power 3.1.9.2 (Heinrich-Heine-University, Dusseldorf, Germany) with an alpha level of 0.05, power established at 0.80, and an effect size of 0.279. The optimal sample size calculated turned out to be 204 in each sample group (control and intervention group), yielding an optimal total sample size of 408. Based on previous survey studies from Norway in similar settings, the expected response rate is 55–70% at each of the measurement times. For the baseline measurement, the expected response rate will provide a sample size in the area of 750–1,050 respondents. Across all three time points, such a response rate will provide a sample size of about 190–515 respondents. Thus, the intervention study will strive for meeting the high end of the expected response rate of similar studies to meet the estimate from the power analysis and should have no problem in meeting sample sized from T1 to T2. The survey will be conducted electronically. With a total of three assessments over 10–12 months, the time interval between the measurement times will be 4 months.

### Questionnaire Instruments

In total, the effects of the intervention will be assessed with a 74-item questionnaire. The questionnaire contains items and inventories that can be classified into the following main categories: (1) demographics and background information, (2) observations of and exposure to ongoing bullying as well as more infrequent acts of bullying and harassment, (3) perceptions of bystander norms, trust in colleagues, and social climate, and (4) perceived behavioral control in the role as bystander.

The majority of the items are taken from established and validated indicators for assessing aspects of the workplace climate and workplace bullying, whereas some were developed for this project in order to fully capture perceived bystander norms and perceived behavioral control related to being a bystander in bullying situations. The items developed for this study is based on literature and/or suggestions from a reference group of specialists on the issue of workplace bullying and harassment. To date, there are no well-established measuring instruments for studying bystander norms and bystander behavioral control, hence such an instrument needed to be developed and tested for the present project. The developed scale has been psychometrically tested and validated in a pilot study of 70 elected health and safety representatives at the case company prior to the intervention project. The other scales have previously been validated in national and international studies.

#### Background Information and Demographics

Background and demographic factors that will be recorded in the survey include age, gender, educational level, position level (employee, manager with personnel responsibility, manager without personnel responsibility, labor union representative, health and safety representative), seniority, and if they have changed work teams during the last 3 months.

#### Bullying and Negative Acts

*Workplace Bullying-*Exposure to bullying behaviors from colleagues in the workplace is measured with the nine-item version of the *Negative Acts Questionnaire-Revised* (NAQ-R) inventory (Notelaers et al., 2018). NAQ-R describes negative and unwanted behaviors that may be perceived as bullying if occurring on a regular basis. All items are formulated in behavioral terms and focus on the mere exposure to inappropriate behaviors while at work with no references to the term bullying (Einarsen and Nielsen, 2015). The scale has proven reliable in former studies, calculating Cronbach’s Alpha for the nine-item version to be 0.86 (Einarsen et al., 2018). In addition, three items from the original version are included, as these three items addresses negative acts related to sexual behavior. Example items are “Spreading of gossip and rumors about you” and “Being shouted at or being the target of spontaneous anger or rage.” The respondents are asked to indicate how often they have been exposed to each specific item in the questionnaire at their present worksite during the last 6 months. Response categories range from 1 to 5 (“never,” “now and then,” “monthly,” “weekly,” and “daily”).

Observed bullying will be measured with the use of the *Bergen Bullying Indicator* (Einarsen and Raknes, 1991), a five-item instrument which measures the degree to which bullying is perceived to constitute a problem at the workplace (e.g., “Bullying at my workplace reduces our efficiency”). The scale has been used in former studies, which has reported Cronbach’s Alpha to be 0.93 (Hauan and Klaveness, 2018). A five-point Likert scale, ranging from totally agree to totally disagree, was applied.

*Observed and witnessed bullying* will also be measured with a single item; “Over the past 4 months, have you observed anyone being bullied in your team/department?”, accompanied by five answer alternatives with regard to frequency of experience (“No, never,” “Yes, a couple of times,” “Yes, every now and then,” “Yes, several times per week,” and “Yes, daily”).

The *observed bullying* question is followed by another question which measure whether or not the respondent intervened if (s)he observed bullying in her/his team. The response categories are as following: “Yes,” “no,” and “I have not observed any bullying activities.”

*Self-labeled victimization* from workplace bullying will be measured with a single item used in several previous studies on bullying (Olweus, 1991; Einarsen and Skogstad, 1996; Solberg and Olweus, 2003; Nielsen et al., 2011). The respondents are asked “*Have you been subjected to bullying at the workplace during the last* 4 *months?*” Response categories range from 1 to 5 (“no,” “rarely,” “now and then,” “once a week,” and “several times a week”).

#### Social Climate at Work

Social climate will be measured by four items from climate for conflict management (Rivlin, 2001), three items from climate for psychological safety (Edmondson, 1999), and three items from Intragroup Conflict (Jehns, 1995). The scale assesses intragroup trust and perceived fairness and trust in the department’s dispute resolution and conflict management procedures, including trust in managers’ conflict management skills. These scales have been used in numerous studies, and researchers have reported the scales as reliable with Cronbach’s alpha of 0.81 for climate for conflict management (Zahlquist et al., 2019), Cronbach’s alpha of 0.86^1^ for climate for psychological safety (Koopmann et al., 2016), and Cronbach’s alpha of 0.92^2^ for intragroup conflict (Jehns, 1995). Example items are “The management handles cases of conflicts well” (conflict management climate), “It is easy to address problems and difficult issues in my work team” (intragroup trust), and “There is jealousy and rivalry between members of my work team” (intragroup conflict). In addition, a five-point Likert scale from 1 (“do not agree”) to 5 (“agree completely”) will be used for responses.

#### Informal Surveillance of Bullying Behaviors

The level of informal surveillance among employees regarding workplace bullying and harassment will be measured with the four-items adapted from an earlier study in Norwegian Municipalities (Hauan and Klaveness, 2018), assessing the awareness, sensitivity, and responsiveness toward workplace bullying among their colleagues. Example items are “If bullying, harassment, or other negative behavior occurs, employees in my team are quick to notice,” and “Employees who see or notice bullying, harassment, or other negative behavior actively monitor whether the behavior develops.” Responses are given on a five-point Likert scale ranging from 1 “incorrect” to 5 “totally correct.” The scale has in prior research yielded a Cronbach’s Alpha at 0.78 (Hauan and Klaveness, 2018).

#### Bystander Norms

Building on the theoretical model of Ajzen (1991), subjective norms will be measured with two scales. The first scale is *Trust in colleagues* and will be assessed with a four-item scale adapted from Cook and Wall (1980). Example items are “If I am exposed to negative acts at work, I know that my colleagues will try to help me” and “If I am exposed to negative acts, I know I will get support and help to deal with bullying and harassment from my colleagues.” A five-point Likert scale from 1 (“completely disagree”) to 5 (“completely agree”) will be used for responses. The scale reliability was analyzed in the data from the pilot study and showed a Cronbach’s Alpha of 0.78.

The second scale is developed for this project and addresses what the respondents believe will be normative reactions/behavior from colleagues when facing bullying situations. The scale hold nine items, and responses are given on a five-point Likert scale ranging from 1 (“incorrect”) to 5 (“totally correct”). Confirmatory factor analysis was applied to the data from the pilot study. The analysis showed two dimensions, one positive bystander behavior and one negative bystander behavior. The positive bystander behavior contains six items and Cronbach’s Alpha is 0.69, and the negative bystander behavior contains three items and Cronbach’s Alpha is 0.66.

#### Attitude

Building on the theoretical model of Ajzen (1991), attitude will be measured with nine-item scale developed for this project. The scale is similar to that of bystander norms developed for this project, however, whereas the former scale measures respondents’ beliefs regarding colleagues normative reactions/behavior when facing bullying situations, the bystander behavior attitude scale measures a persons’ own usual behavior in bullying situations. An example of one item is “If someone is exposed to negative actions in our team, I may be an active participant in these negative actions myself” (normative behavior from the respondent). Responses are given on a five-point Likert scale ranging from 1 (“incorrect”) to 5 totally (“correct”). Again, confirmatory factor analysis was applied to the data from the pilot study. The analysis showed two dimensions, one constructive attitude bystander and one destructive bystander behavior. The constructive attitude bystander behavior contains six items and Cronbach’s Alpha is 0.89. The destructive attitude bystander behavior contains three items and Cronbach’s Alpha is 0.81.

#### Perceived Behavioral Control

Building further on the theoretical model of Ajzen (1991), Perceived behavioral control will be measured with an eight-item scale developed for this project. All items are formulated in behavioral terms and focus on the respondents’ perception of their own ability to handle bullying situations in their team. Example items are “I feel confident in how to handle and intervene in ‘bullying situations’ when they occur” and “I will be able to intervene there and then, if I observe someone being exposed to negative actions by others.” A five-point Likert scale from 1 (“do not agree”) to 5 (“agree completely”) will be used for responses. The scale was tested on the data from the pilot study and Cronbach’s Alpha is 0.86.

### Qualitative Interviews

In the qualitative interview, the selected participants (supervisors, health and safety representatives, and labor union representatives) will be asked to indicate to what extent they think the intervention has had an effect or not, why or why not; and if having an effect, how it has had an effect. More precisely, the qualitative semi-structured focus group interview will be based on the following interview guide:

1. How relevant did you find this intervention in your organization and working environment?
2. What do you see as the perceived strengths and limitations of the intervention?
3. How effective did you find the intervention when it comes to preventing workplace bullying and harassment in your own department?
4. If you found the intervention to be effective, what are the perceived mechanisms that make it effective?
5. Are there mechanisms in the intervention, the sessions, or the organization that may act so as to prevent the intervention form being effective?

The last questions will be analyzed to the extent that they talk about mechanisms in the TPB or if they mention other mechanisms. The qualitative focus group interview will be recorded and transcribed. The data will be analyzed using the NVivo 12.1 computer software package.

### Quantitative Data Analysis and Statistics

All variables in the questionnaire will be measured at all times. The dependent variables can be categorical, continuous, time independent, or time dependent. Data will therefore be analyzed using statistical methods adapted to this type of design and variables. Data will be analyzed with SPSS 25.0, Stata 15, MPlus 8.3, and MLwiN 3.04. Following the described aims, this project will determine prevalence rates; group differences; and direct, indirect, and conditional associations between the study variables both cross-sectionally and over time. Group differences will be tested with chi-square tests and ANOVA. Associations between variables will be tested with correlation and regression-based approaches. Indirect and conditional effects will be analyzed with the process script (Hayes, 2013) and structural equation models. Longitudinal associations between variables will be adjusted for stability in variables in order to model changes over time. Latent class analyses will be used to identify latent profiles of exposure and outcome variables among the respondents (Vermunt and Magidson, 2002; Christensen et al., 2018). In order to capture the multilevel structure of the quantitative study data where the measurements (Level 1) of the study constructs are nested within individuals (Level 2), multilevel analyses will be carried out using MLwiN or Mplus depending on the complexity of hypothesized models. Potential control variables and confounders for the adjusted models, e.g., demographic variables, will be considered only when theoretically applicable (Spector and Brannick, 2011).

## Discussion

This project will address some important knowledge gaps in research on workplace interventions against workplace bullying, focusing on the role of bystanders in a preventive intervention. First, we refine and test a relatively short and focused intervention that may easily be implemented in many organizational contexts. Based on qualitative group interviews, there is reason to believe that such an intervention at least will be highly appreciated by those involved (Mikkelsen and Hogh, 2019). Second, we will evaluate this intervention employing a range of proximal and distal outcomes, such as participant satisfaction, prevalence of bullying both observed and experienced, changes in perceived social climate regarding interpersonal trust, and conflict management. Based on the TPB, we will particularly look at changes in attitudes, perceived behavioral control, and social norms as possible proximal and mediating factors. Third, whereas previous studies on interventions on workplace bullying have mainly used qualitative methods or inadequate designs based on quantitative methods (Escartin, 2016; Gillen et al., 2017), the present project is based on an experimental RCT design including longitudinal quantitative survey data, which is a novel approach within this field of research. Fourth, by examining mediating and moderating variables in the relationship between perceived bystander behavior control and outcomes, this project will generate novel knowledge important for extending and developing the theoretical basis of our understanding of bystander behavior, which is critical in order to adequately design upcoming studies and further interventions in this area. From an applied perspective, the current project will elucidate specific prevention and management strategies that can help organizations protect their employees against the detrimental effects of workplace bullying. The resulting knowledge will aid efforts to improve employee well-being and the overall quality of work environment. Knowledge about preventive measures against bullying among employees is important in order to protect the health, well-being, and work ability of employees, which in turn will determine the quality of the work they perform. The results of this project will help identify whether/how specific bullying prevention and harassment measures at work are associated with decreases in workplace bullying prevalence. Moreover, it will shed light on the mechanisms that may explain why such an intervention may work or not.

## Strengths and Limitations

The study and its design have several strengths. Through a longitudinal, RCT design, this project performs repeated measurements of social work environmental factors (climates, norms, and trust), bystander behavioral control, intervening variables, and outcomes. This provides more reliable data not only on the possible outcomes of the intervention, yet also on providing possible explanations and moderating psychological factors related to the perceptions, cognitions, emotions, and behaviors of bystanders. As conducting and measuring effects of intervention study on workplace bullying is difficult (Escartin, 2016), prospective designs are the strongest form of scientific evidence for the causal association between organizational interventions and potential effects. Furthermore, the survey builds mainly on well-established and standardized inventories, psychometrically tested for validity and reliability or scales that are tailor-made, yet tested for the present project. The data structure allows for multilevel models where individual level data are aggregated to work units/organizational levels. Depending on the participation, it is likely that the sample will be representative for the case company.

There are also some limitations of the planned project. The included survey instruments are all self-report measures, and the project is thus subject to limitations specific to self-report instruments such as response-set tendencies. The survey data are also measured from the same source, yet at different time points. As such, common method variance may inflate the relations between constructs somewhat (Podsakoff et al., 2003). However, the use of a longitudinal design as well as the opportunity to obtain co-worker reports of working conditions at the team and work unit levels should limit the risk of common method variance caused by self-report biases. Regardless, the main aim of the present study is exactly that, to investigate if a given intervention will change the perceptions of workers regarding their own perceived exposure to bullying, as well as the potential support one may receive from colleagues.

### Assessment of Potential Bias

#### Response Bias

Due to their personal experiences, employees may be more inclined to participate in the survey and intervention if they have been exposed to or observed harassment and bullying at the workplace. This situation is likely to inflate the prevalence estimates found in our sample in case of substantial non-response among non-exposed workers. We attempt to mitigate this problem by informing the respondents of their value to the study even though they have not experienced or witnessed bullying. To motivate all organizational members to participate, information about the utility of the intervention “Grip inn/Intervene” as a tool for each work unit to assess and improve working conditions will be carefully disseminated to all key personnel. This will be ensured by holding information meetings with all managers, foremen, labor union representatives, and health and safety representatives, during which there will be an emphasis on their roles as motivators for their colleagues. Furthermore, this information will also be communicated through the case company’s intranet. To reinforce commitment in the organization, the head executive vice-president will send out information and encouragement to participate in the survey and intervention, prior to the distribution of the survey. Moreover, up to four reminders will be sent during the survey collection period. These reminders will be sent by both the researchers and managers^3^. The strict procedures for confidential treatment of data will also be highlighted.

#### Recall Bias

As is common with questionnaire surveys asking about a person’s past experiences, there is a risk of recall bias. To minimize this risk, we have included a relatively short and specific time period for most items (last 4 months before the survey), and items have a relatively low level of abstraction and should therefore be likely to be associated with specific events. As bullying represents a violation of a person’s physical and psychological integrity, people are likely to remember such events.

#### Selection Bias

As all relevant employees in the case company will be invited to participate in the survey, there is no risk of selection bias due to inclusion criteria defined by the researchers.

#### Contamination of Control Group

As the main location for the intervention study will be divided into intervention groups and control groups, there is a risk that the project may also have effects in the control groups. This is particularly the case as we may need to work quite a bit with the whole organization to secure a high participation rate in the organization. To counteract this problem, we will use location B as a second control group. Apart from a managerial level, there is little interaction between employees in the two locations of this organization.

### Dissemination

The results of the study will be submitted to and presented in international peer-reviewed scientific journals. Results will also be presented at national and international scientific conferences and other types of seminars. The company used as the case study will be informed about the findings through a series of feedback meetings. The project is important for the case company, as they will use the findings from the intervention to decide whether the measure/intervention will be implemented throughout the organization. The case company has no clauses in relation to publication of results, except that they will consider whether they will be presented by name in these publications or referred to as a Norwegian industrial company in the oil/offshore industry.

## Conclusion

Given the scarcity of evidence on effective interventions for preventing and managing workplace bullying, this study is important and timely and may help enhance our knowledge on how organizations may focus their efforts and resources in combating workplace bullying. The project should thereby inform organizations with regard to actions in the form of intervention programs.

## Data Availability Statement

The original contributions presented in the study are included in the article/Supplementary Material and further inquiries can be directed to the corresponding author.

## Ethics Statement

In line with the General Data Protection Regulation (GDPR), the project has acquired permission from the Norwegian Centre for Research Data (NSD; approval: 226309) to process the personal data in this project for research purposes. Since the project does not include health data, approval from the Regional Ethics Committee is not required. Department of Psychosocial Science, UiB, is subject to the Personal Data Act regarding proper processing of personal data. Participation in the data collection will be voluntary and based on informed consent of the respondents. Data will be treated confidentially and no individuals will be identified in the presentation of project results. Potential risks for participants in this study include psychological and social harm due to the potential of participants recalling traumatic incidents which may cause anxiety and the possibility that conflicts will play out in the sessions. These risks were evaluated and identified as being minimal due to the planned structure and content of the intervention and due to the fact that the said organization has proper procedures to handle such situations. The intervention is a typical work environment intervention where one discusses one’s own work environment and how this can be improved by managers and colleagues intervening in risk situations. The data will be completely anonymized at the end of the project. The project will be finished by December 31, 2024. All participants who are included in the questionnaire survey will sign an informed consent before participation.

## Author Contributions

All authors contributed to the development and refinement of the intervention and/or the research design. KE, MN, and SE were responsible for the writing of the manuscript. All authors contributed to the article and approved the submitted version.

### Conflict of Interest

JK was employed by the company Kvaerner AS. SE has prior to this project been a consultant for the said company when they developed their complaints procedures. No such role exists in relation to the present project.

The remaining authors declare that the research was conducted in the absence of any commercial or financial relationships that could be construed as a potential conflict of interest.

## Footnotes

^1^The Cronbach’s alpha in the study of Koopmann et al. (2016) contains the original scale of seven items. The present study contains three items from the scale.

^2^The Cronbach’s alpha in the study of Jehns (1995) is of task related conflicts.

^3^The reminders from the researcher and managers will be coordinated where the managers’ reminders will be sent 15 min before the researchers send the reminder containing an individual link to the electronic questionnaire.
