# Supplementary material for: Outcomes of a Proximal Workplace Intervention Against Workplace Bullying and Harassment: A Protocol for a Cluster Randomized Controlled Trial Among Norwegian Industrial Workers
Source: Front Psychol. 2020 Aug 31;11:2013. doi: 10.3389/fpsyg.2020.02013 (PMC7489146; doi:10.3389/fpsyg.2020.02013)
Supplement: Supplementary file 1 [file Data_Sheet_1.PDF]

Survey on the occurrence and the handling of bullying and harassment

The University of XXX and XXXXXXX have entered a collaboration to implement and evaluate a work environment intervention aimed at reducing bullying and harassment in working life. In addition to evaluating whether the measure works, the results of the study will be a part of the research at the Department of Psychosocial Science, XXX. The aim of the project is to study a) how employees experience and assess the effectiveness of the intervention "Invervene!", and b) how the intervention may contribute to lower frequencies of bullying and harassment as well as better ability to handle incidents of bullying situations of employees and managers.

Why are you asked to participate?

You are asked to participate because you are employed by a Location A or Location B, where the company and the employees' representatives have wanted to implement this interventon.

If you want to read more about participation and privacy, click here.

If you have any questions about the project, please contact XXXXXXXXXXX

Privacy officer at the institution is xxxxx.

Background information and demographics

<u>Are you ....?</u>

(1) Male

(2) Female

(3) Other

<u>What is your job in Kvaerner?</u>

(5) apprenticeship

(1) Operator

(2) Official

(3) Manager without staff responsibility

(4) Manager with personnel responsibility

<u>Do you also hold any special position?</u>

(1) No, none

(2) Yes, elected representative

(3) Yes, safety delegate

(4) Yes, both union representative and safety representative

How long have you worked in the organisation?

(1) Less than 1 year

(2) 1-5 years

(3) 6-10 years

(4) 11-20 years

(5) over 20 years

In the last 4 months, have you changed department?

(1) Yes

(2) No

What is your highest completed education?

(1) Elementary

(2) High school / vocational school

(3) certificate of apprenticeship

(4) College / University lower degree (Year of study / Bachelor's degree)

(5) College / University higher degree (Master's degree / PhD)

12.6 Age. Enter the age in the box below

______

Bullying and negative acts

About bullying and harassment in working life:

Bullying and harassment is a collective term for various negative and offensive acts in the work environment. These actions can occur in various forms and in varying degrees of severity and are more or less systematically aimed at one or more persons. Examples of such negative actions are someone talking behind one's back, being ridiculed or ridiculed in a disrespectful way, being excluded from the social community, or being subjected to unwanted sexual attention.

Negative acts questionnaire (NAQ-R)

How often have you been exposed to the following unwanted actions or negative situations in your workplace in the last 4 months?

(1. Never) (2. Occasionally) (3. Monthly) (4. Weekly) (5. Daily)

Someone withholding information which affects your performance

Being ignored or facing a hostile reaction when you approach

Spreading of gossip and rumors about you

Being ignored or excluded

Having insulting or offensive remarks made about your person, your attitudes, or your private life

Being shouted at or being the target of spontaneous anger

Repeated reminders of your errors or mistakes

Persistent criticism of your errors or mistakes

Practical jokes carried out by people you don't get along with

Unwanted sexual approaches that you experienced as unpleasant

Sexually charged stare or other unpleasant looks

Unwanted physical contact with sexual undertones, such as clapping, pinching, itching or embracing

Have you been bullied in your workplace for the past 4 months?

(1) No

(2) Yes, a rare occasion

(3) Yes, sometimes

(4) Yes, several times a week

(5) Yes, daily

Bergen Bullying Indicator (BBI)

Consider the following statements:

Bullying and Harassment ...

1 "incorrect" to 5 "totally correct"

… reduce the job satisfaction of many employees in my department

… reduce the efficiency of my department

… constitute a serious stressor for many in my department

… leads to poor cooperation in my department

… is a problem in my department

… occur in my department

In the last 4 months have you experienced that someone has been bullied in your department?

(1) No

(2) Yes, a rare occasion

(3) Yes, sometimes

(4) Yes, several times a week

(5) Yes, daily

If you have observed bullying in your work group / department, did you intervene in any way?

(1) Yes

(2) No

(3) I have not observed bullying at all in our department

Social Climate at work

The claims revolve around how employees at your workplace perceive that conflicts and bullying / harassment in the work environment are generally handled:

(1. Totally Wrong) (2. Wrong) (3. Neither Wrong nor Right) (4. Right) (5. Totally Right)

Conflict management climate:

If I have a serious disagreement with someone at work, I know who I should talk to about it

The way we deal with disagreements between employees in my unit works well

My superiors deal with conflicts in a good manner

We have good procedures and methods for raising disagreements and conflicts in my workplace

Intragroup Conflict:

In this department it is safe to address problems and difficult.

There are much interpersonal tension between members in our department

There are generally much friction among members in our department

Psychological safety:

Occasionally people in my department get angry in connection with work

There are jealousy and rivalry between members in our department

It is easy to ask other members of this team for help in our department

Informal surveillance of bullying behaviors

In the following, you will be asked statements about how colleagues notice situations in which bullying can occur.

How do you feel that these statements fit your working day?

1 “incorrect” to 5 “totally correct”

If bullying or harassment occurs, employees in my department are quick to notice

Employees in my department who see or notice bullying or harassment will address the perpetrator

Employees who observe bullying or harassment will actively monitor the situation to see if the behavior develops

In our department we follow closely how we behave towards each other

Bystander norms

Trust in colleagues

How much do you agree or disagree with the following statements about your relationship with your colleagues regarding these types of situations?

(1. Strongly Disagree) (2. Disagree) (3. Neither agree nor disagree) (4. Agree) (5. Strongly agree)

If exposed to bullying behavior at work, I know that my work colleagues will try to help me

I can trust that most of my work colleagues are reliable and intervene when they see others being treated poorly

I have full confidence in my work colleagues' skills in dealing with the bullying behavior of others

If subjected to bullying behavior, I will receive support and assistance in dealing with this bullying behavior from my work colleagues

How do you find colleagues in your work group / department acting in situations where someone is subjected to bullying, harassment, or other negative actions?

If someone is exposed to bullying behavior in our department, it happens that…

1 “incorrect” to 5 “totally correct”

… there are colleague(s) other than the perpetrator that orchestra the bullying situation and "pulls the threads"

… one or more colleague(s) are themselves actively involved in these bullying behaviors

… colleague(s) are inclined to withdraw from the situation where these bullying behaviors takes place

… colleague(s) try to calm down the situation

… colleague(s) intervenes in the situation and defends the target

… colleague(s) try to stop the perpetrator

… colleague(s) try to talk to both parties to end the bullying situation

… colleague(s) express that they do not like the actions of the person who directly performs the bullying behaviors

… colleague(s) easily join in on the bullying

Attitude

If we think about it, we have all been in situations where someone is subjected to bullying, harassment or other negative acts. To be perfectly honest, how do you usually act in these kinds of situations?

If someone is subjected to negative acts on our work group / department it happens that I ...

1 “incorrect” to 5 “totally correct”

… am the one behind it all and who "pulls the threads"

… actively participate in these negative actions myself

… withdraw from the situation where these negative actions are taking place

… try to calm down the situation

… provide support in hindsight to those who were targeted

… defend the person exposed to the negative actions

… try to stop the person performing the negative actions

… try to talk to both parties to end the bullying situation

… clearly state that I do not like the actions of the perpetrator

… try to switch conversation topic to end negative actions

… can even be pulled in on and contribute to more negative actions myself

Perceived behavioral control

In situations where colleagues are exposed to or subject others to negative actions, it can be difficult to know for yourself how to react.

How is this for you at the moment?

(1. Strongly Disagree) (2. Disagree) (3. Neither agree nor disagree) (4. Agree) (5. Strongly agree)

I clearly see how I can intervene and counter "bullying situations" if I see a colleague being bullied

I can handle bullying and harassment situations in my work group / department because I am able to intervene in the situation

I feel confident in how to handle and intervene in "bullying situations" when they occur

I can easily identify "bullying situations" that may occur in the work group / in my department

If I observe someone who exposes others to negative actions, I feel able to intervene there and then

I can see how bullies choose their victims

I can handle the situation if I am bullied by a colleague

I am able to communicate in an acceptable manner if I confront a colleague who has been bullied

I clearly see what to do if I am exposed to negative actions

I am sure that I will be able to counteract if I experience being subjected to negative actions
